# Supplementary material for: More Rule than Exception: Parallel Evidence of Ancient Migrations in Grammars and Genomes of Finno-Ugric Speakers
Source: Genes (Basel). 2020 Dec 11;11(12):1491. doi: 10.3390/genes11121491 (PMC7763979; doi:10.3390/genes11121491)
Supplement: Supplementary file 1 [file genes-11-01491-s001.pdf]

Supplementary Material.

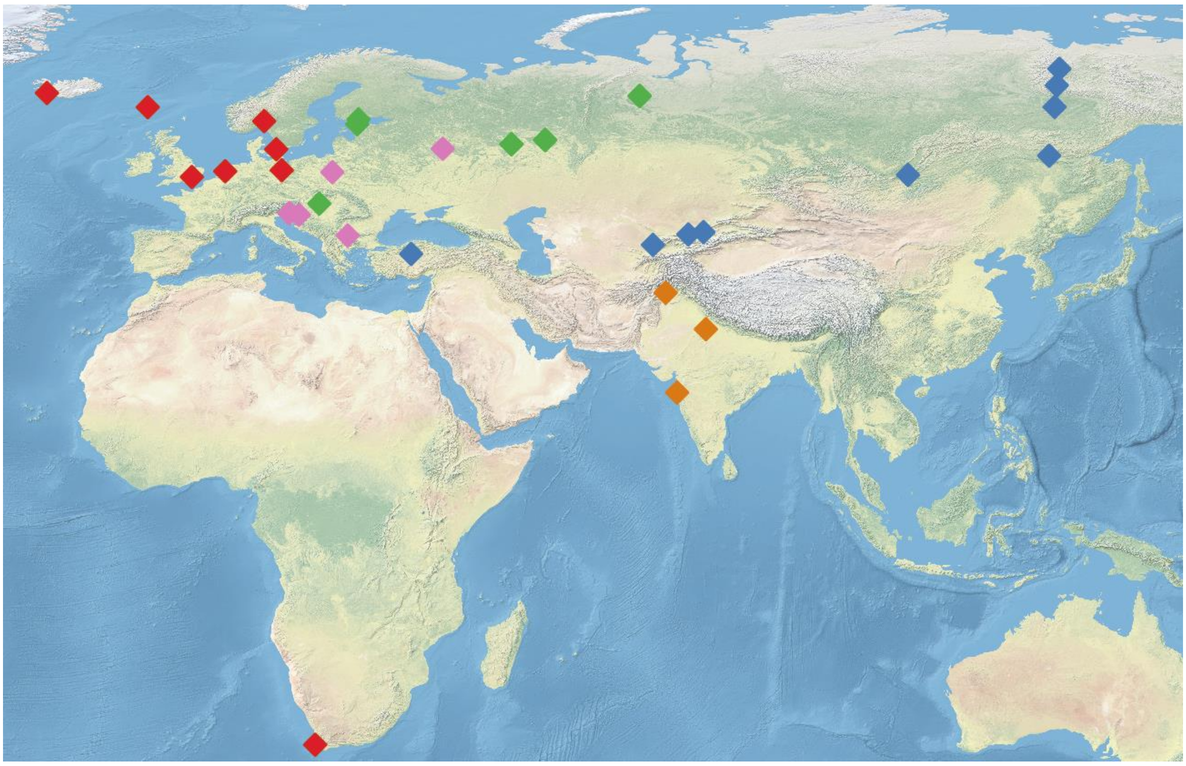

Supplementary Figure S1. Approximate geographical location of the 34 languages considered. Thirty-one geographical points; three FU languages, Mari, Udmurt and Khanty, are represented each by two diastratic variants spoken in the same location. Language groups coded as follows: Finno-Ugric (green); Altaic (blue); Germanic (red); Slavic (pink); Indo-Iranian (orange).

	English	Dutch	Afrikaans	German	Danish	Icelandic	Faroese	Norwegian	Bulgarian	Serbo-Croatian	Slovenian
English	0	0.125	0.087	0.16	0.12	0.192	0.12	0.12	0.231	0.261	0.261
Dutch	0.125	0	0.043	0.042	0.12	0.154	0.12	0.12	0.192	0.217	0.217
Afrikaans	0.087	0.043	0	0.083	0.16	0.192	0.16	0.16	0.231	0.261	0.261
German	0.16	0.042	0.083	0	0.154	0.115	0.154	0.154	0.222	0.174	0.174
Danish	0.12	0.12	0.16	0.154	0	0.111	0.038	0.074	0.214	0.217	0.217
Icelandic	0.192	0.154	0.192	0.115	0.111	0	0.143	0.107	0.2	0.174	0.174
Faroese	0.12	0.12	0.16	0.154	0.038	0.143	0	0.037	0.241	0.217	0.217
Norwegian	0.12	0.12	0.16	0.154	0.074	0.107	0.037	0	0.207	0.217	0.217
Bulgarian	0.231	0.192	0.231	0.222	0.214	0.2	0.241	0.207	0	0.125	0.125
Serbo-Croatian	0.261	0.217	0.261	0.174	0.217	0.174	0.217	0.217	0.125	0	0
Slovenian	0.261	0.217	0.261	0.174	0.217	0.174	0.217	0.217	0.125	0	0
Polish	0.261	0.217	0.261	0.208	0.217	0.208	0.217	0.217	0.167	0.087	0.087
Russian	0.261	0.217	0.261	0.208	0.217	0.208	0.217	0.217	0.125	0.043	0.043
Marathi	0.35	0.3	0.35	0.333	0.3	0.333	0.3	0.3	0.333	0.35	0.35
Hindi	0.316	0.263	0.316	0.3	0.263	0.3	0.263	0.263	0.3	0.316	0.316
Pashto	0.316	0.263	0.316	0.3	0.263	0.263	0.263	0.222	0.263	0.35	0.35
Hungarian	0.5	0.545	0.524	0.545	0.565	0.565	0.565	0.565	0.545	0.55	0.55
Khanty_1	0.529	0.556	0.529	0.556	0.556	0.556	0.556	0.556	0.579	0.556	0.556
Khanty_2	0.5	0.526	0.5	0.526	0.526	0.526	0.526	0.526	0.55	0.526	0.526
Estonian	0.412	0.444	0.412	0.444	0.444	0.444	0.444	0.444	0.5	0.474	0.474
Finnish	0.474	0.5	0.474	0.5	0.5	0.5	0.5	0.5	0.524	0.5	0.5
Mari_1	0.471	0.5	0.471	0.5	0.5	0.5	0.5	0.5	0.526	0.5	0.5
Mari_2	0.444	0.474	0.444	0.474	0.474	0.474	0.474	0.474	0.5	0.474	0.474
Udmurt_1	0.5	0.526	0.5	0.526	0.526	0.526	0.526	0.526	0.55	0.526	0.526
Udmurt_2	0.474	0.5	0.474	0.5	0.5	0.5	0.5	0.5	0.524	0.5	0.5
Even_1	0.474	0.5	0.474	0.5	0.5	0.5	0.5	0.5	0.5	0.474	0.474
Even_2	0.474	0.5	0.474	0.5	0.5	0.5	0.5	0.5	0.5	0.474	0.474
Evenki	0.474	0.5	0.474	0.5	0.5	0.5	0.5	0.5	0.5	0.474	0.474
Yakut	0.5	0.526	0.5	0.526	0.526	0.526	0.526	0.526	0.526	0.5	0.5
Uzbek	0.438	0.471	0.438	0.471	0.471	0.471	0.471	0.471	0.471	0.438	0.438
Kazakh	0.471	0.5	0.471	0.5	0.5	0.5	0.5	0.5	0.5	0.471	0.471
Kirghiz	0.471	0.5	0.471	0.5	0.5	0.5	0.5	0.5	0.5	0.471	0.471
Turkish	0.471	0.5	0.471	0.5	0.5	0.5	0.5	0.5	0.5	0.471	0.471
Buryat	0.474	0.5	0.474	0.524	0.5	0.524	0.5	0.5	0.5	0.55	0.55

	Polish	Russian	Marathi	Hindi	Pashto	Hungarian	Khanty_1	Khanty_2	Estonian	Finnish	Mari_1	Mari_2
English	0.261	0.261	0.35	0.316	0.316	0.5	0.529	0.5	0.412	0.474	0.471	0.444
Dutch	0.217	0.217	0.3	0.263	0.263	0.545	0.556	0.526	0.444	0.5	0.5	0.474
Afrikaans	0.261	0.261	0.35	0.316	0.316	0.524	0.529	0.5	0.412	0.474	0.471	0.444
German	0.208	0.208	0.333	0.3	0.3	0.545	0.556	0.526	0.444	0.5	0.5	0.474
Danish	0.217	0.217	0.3	0.263	0.263	0.565	0.556	0.526	0.444	0.5	0.5	0.474
Icelandic	0.208	0.208	0.333	0.3	0.263	0.565	0.556	0.526	0.444	0.5	0.5	0.474
Faroese	0.217	0.217	0.3	0.263	0.263	0.565	0.556	0.526	0.444	0.5	0.5	0.474
Norwegian	0.217	0.217	0.3	0.263	0.222	0.565	0.556	0.526	0.444	0.5	0.5	0.474
Bulgarian	0.167	0.125	0.333	0.3	0.263	0.545	0.579	0.55	0.5	0.524	0.526	0.5
Serbo-Croatian	0.087	0.043	0.35	0.316	0.35	0.55	0.556	0.526	0.474	0.5	0.5	0.474
Slovenian	0.087	0.043	0.35	0.316	0.35	0.55	0.556	0.526	0.474	0.5	0.5	0.474
Polish	0	0.043	0.35	0.316	0.35	0.55	0.556	0.526	0.474	0.5	0.5	0.474
Russian	0.043	0	0.35	0.316	0.35	0.55	0.556	0.526	0.474	0.5	0.5	0.474
Marathi	0.35	0.35	0	0.05	0.19	0.5	0.471	0.389	0.474	0.45	0.389	0.316
Hindi	0.316	0.316	0.05	0	0.238	0.474	0.471	0.444	0.444	0.5	0.389	0.368
Pashto	0.35	0.35	0.19	0.238	0	0.55	0.529	0.444	0.474	0.421	0.5	0.421
Hungarian	0.55	0.55	0.5	0.474	0.55	0	0.188	0.176	0.312	0.333	0.235	0.222
Khanty_1	0.596	0.556	0.471	0.471	0.529	0.188	0	0.067	0.4	0.353	0.188	0.235
Khanty_2	0.526	0.526	0.389	0.444	0.444	0.176	0.067	0	0.375	0.278	0.235	0.167
Estonian	0.474	0.474	0.474	0.444	0.474	0.312	0.4	0.375	0	0.125	0.267	0.25
Finnish	0.5	0.5	0.45	0.5	0.421	0.333	0.353	0.278	0.125	0	0.235	0.167
Mari_1	0.5	0.5	0.389	0.389	0.5	0.235	0.188	0.235	0.267	0.235	0	0.099
Mari_2	0.474	0.474	0.316	0.368	0.421	0.222	0.235	0.167	0.25	0.167	0.059	0
Udmurt_1	0.526	0.526	0.421	0.421	0.526	0.235	0.188	0.235	0.25	0.222	0.059	0.111
Udmurt_2	0.5	0.5	0.35	0.4	0.45	0.222	0.235	0.167	0.235	0.158	0.111	0.053
Even_1	0.474	0.474	0.3	0.3	0.4	0.368	0.389	0.421	0.412	0.421	0.222	0.263
Even_2	0.474	0.474	0.3	0.3	0.4	0.368	0.389	0.421	0.412	0.421	0.222	0.263
Evenki	0.474	0.474	0.3	0.3	0.4	0.368	0.389	0.421	0.412	0.421	0.222	0.263
Yakut	0.5	0.5	0.316	0.316	0.421	0.294	0.2	0.25	0.438	0.444	0.235	0.278
Uzbek	0.438	0.438	0.278	0.278	0.389	0.353	0.267	0.312	0.4	0.412	0.188	0.235
Kazakh	0.471	0.471	0.278	0.278	0.389	0.353	0.267	0.312	0.4	0.412	0.188	0.235
Kirghiz	0.471	0.471	0.278	0.278	0.389	0.353	0.267	0.312	0.4	0.412	0.188	0.235
Turkish	0.471	0.471	0.278	0.278	0.389	0.353	0.267	0.312	0.4	0.412	0.188	0.235
Buryat	0.55	0.55	0.35	0.35	0.45	0.389	0.312	0.353	0.444	0.421	0.278	0.316

	Udmurt_1	Udmurt_2	Even_1	Even_2	Evenki	Yakut	Uzbek	Kazakh	Kirghiz	Turkish	Buryat
English	0.5	0.474	0.474	0.474	0.474	0.5	0.438	0.471	0.471	0.471	0.474
Dutch	0.526	0.5	0.5	0.5	0.5	0.526	0.471	0.5	0.5	0.5	0.5
Afrikaans	0.5	0.474	0.474	0.474	0.474	0.5	0.438	0.471	0.471	0.471	0.474
German	0.526	0.5	0.5	0.5	0.5	0.526	0.471	0.5	0.5	0.5	0.524
Danish	0.526	0.5	0.5	0.5	0.5	0.526	0.471	0.5	0.5	0.5	0.5
Icelandic	0.526	0.5	0.5	0.5	0.5	0.526	0.471	0.5	0.5	0.5	0.524
Faroese	0.526	0.5	0.5	0.5	0.5	0.526	0.471	0.5	0.5	0.5	0.5
Norwegian	0.526	0.5	0.5	0.5	0.5	0.526	0.471	0.5	0.5	0.5	0.5
Bulgarian	0.55	0.524	0.5	0.5	0.5	0.526	0.471	0.5	0.5	0.5	0.5
Serbo-Croatian	0.526	0.5	0.474	0.474	0.474	0.5	0.438	0.471	0.471	0.471	0.55
Slovenian	0.526	0.5	0.474	0.474	0.474	0.5	0.438	0.471	0.471	0.471	0.55
Polish	0.526	0.5	0.474	0.474	0.474	0.5	0.438	0.471	0.471	0.471	0.55
Russian	0.526	0.5	0.474	0.474	0.474	0.5	0.438	0.471	0.471	0.471	0.55
Marathi	0.421	0.35	0.3	0.3	0.3	0.316	0.278	0.278	0.278	0.278	0.35
Hindi	0.421	0.4	0.3	0.3	0.3	0.316	0.278	0.278	0.278	0.278	0.35
Pashto	0.526	0.45	0.4	0.4	0.4	0.421	0.389	0.389	0.389	0.389	0.45
Hungarian	0.235	0.222	0.368	0.368	0.368	0.294	0.353	0.353	0.353	0.353	0.389
Khanty_1	0.188	0.235	0.389	0.389	0.389	0.2	0.267	0.267	0.267	0.267	0.312
Khanty_2	0.235	0.167	0.421	0.421	0.421	0.25	0.312	0.312	0.312	0.312	0.353
Estonian	0.25	0.235	0.412	0.412	0.412	0.438	0.4	0.4	0.4	0.4	0.444
Finnish	0.222	0.158	0.421	0.421	0.421	0.444	0.412	0.412	0.412	0.412	0.421
Mari_1	0.059	0.111	0.222	0.222	0.222	0.235	0.188	0.188	0.188	0.188	0.278
Mari_2	0.111	0.053	0.263	0.263	0.263	0.278	0.235	0.235	0.235	0.235	0.316
Udmurt_1	0	0.053	0.263	0.263	0.263	0.278	0.235	0.235	0.235	0.235	0.263
Udmurt_2	0.053	0	0.3	0.3	0.3	0.316	0.278	0.278	0.278	0.278	0.3
Even_1	0.263	0.3	0	0	0	0.211	0.167	0.167	0.167	0.167	0.316
Even_2	0.263	0.3	0	0	0	0.211	0.167	0.167	0.167	0.167	0.316
Evenki	0.263	0.3	0	0	0	0.211	0.167	0.167	0.167	0.167	0.316
Yakut	0.278	0.316	0.211	0.211	0.211	0	0.118	0.062	0.062	0.062	0.235
Uzbek	0.235	0.278	0.167	0.167	0.167	0.118	0	0.062	0.062	0.062	0.235
Kazakh	0.235	0.278	0.167	0.167	0.167	0.062	0.062	0	0	0	0.188
Kirghiz	0.235	0.278	0.167	0.167	0.167	0.062	0.062	0	0	0	0.188
Turkish	0.235	0.278	0.167	0.167	0.167	0.062	0.062	0	0	0	0.188
Buryat	0.263	0.3	0.316	0.316	0.316	0.235	0.235	0.188	0.188	0.188	0

Supplementary Figure S2. Syntactic distances.

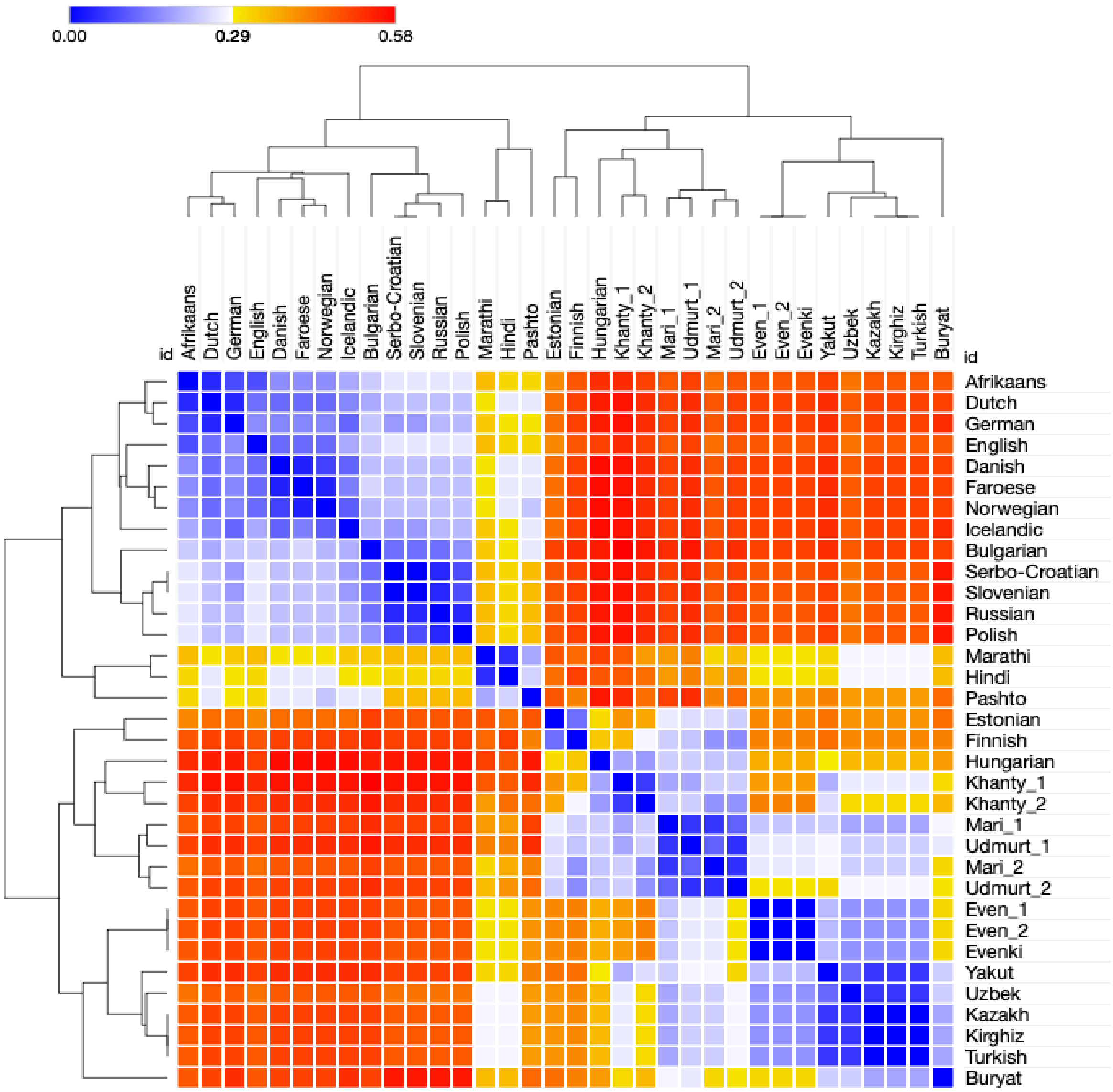

Supplementary Figure S3. Heatmap from the syntactic distances. Dark red represents maximum distance, dark blue minimum distance.

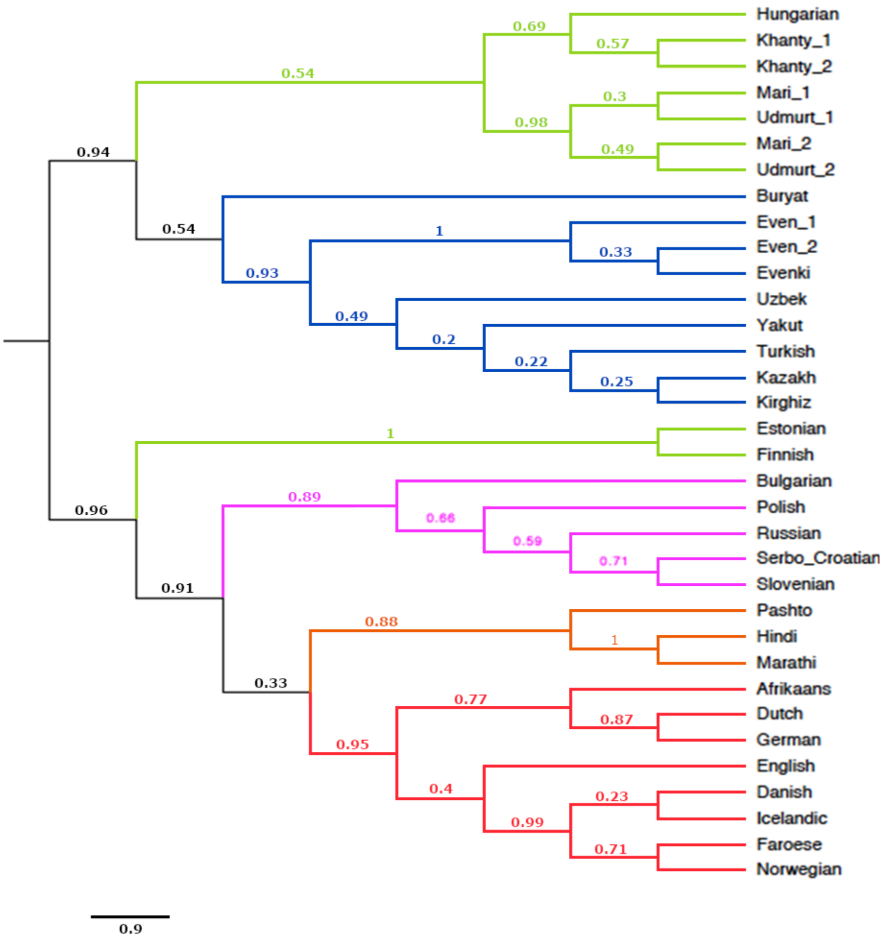

Supplementary Figure S4. Bayesian phylogeny (BEAST) from the syntactic dataset. Numbers on the nodes represent the posterior probability. Orange=Indo-Iranian IE, pink=Slavic IE, red=Germanic IE, Blue=AL, Green=FU.

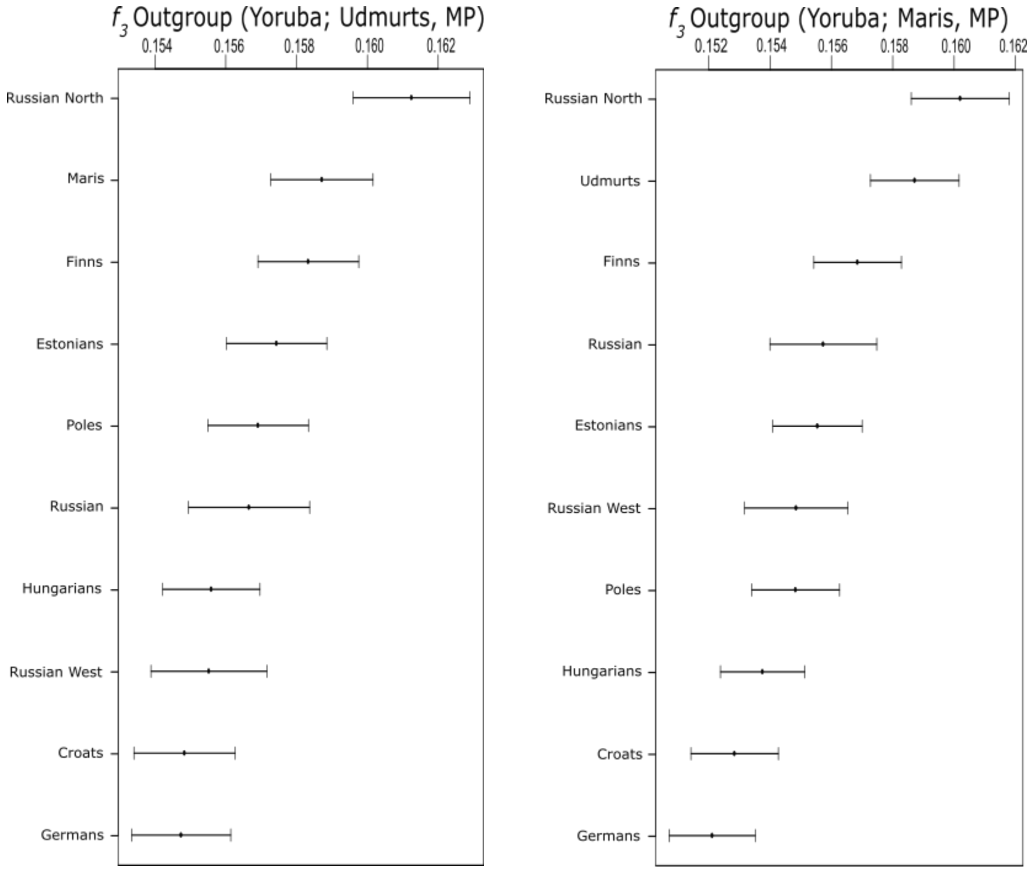

Supplementary Figure S5. Outgroup *f3*-statistics analysis. Shared genetic drift between modern Pontic steppes populations and modern European populations (MP).

Supplementary Table S1. Whole-genome samples collected for the populations under study.

Sample Size	Sample ID	Populations	Country	Region	Coverage	Language Family	Reference
3	Est1, Est2, Est3	**Estonian**	Estonia	Europe	>40	**Uralic**	Pagani *et al.* 2016
3	Fin1, Fin2, Fin3	**Finnish**	Finland	Europe	>40	**Uralic**	Pagani *et al.* 2016
3	Rus1, RusPi1, RusPs2	**Russian (North and West)**	Russia	Europe	>40	**Indo-European**	Pagani *et al.* 2016
3	Pole1, Pole2, Pole3	**Polish**	Poland	Europe	>40	**Indo-European**	Pagani *et al.* 2016
3	Hun1, Hun2, Hun4	**Hungarian**	Hungary	Europe	>40	**Uralic**	Pagani *et al.* 2016
3	Ger1, Ger2, Ger3	**German**	Germany	Europe	>40	**Indo-European**	Pagani *et al.* 2016
3	croat11, croat13, croat12	**Croatian**	Bosnia-Herzegovina	Europe	>40	**Indo-European**	Pagani *et al.* 2016
3	Iran1, Iran2, Iran3	**Iranian (Farsi)**	Iran	West Asia	>40	**Indo-European**	Pagani *et al.* 2016
3	Mari1, Mari2, Mari3	**Mari**	Russia	Europe	>40	**Uralic**	Pagani *et al.* 2016
3	Udmrd1, Udmrd2, Udmrd3	**Udmurt**	Russia	Europe	>40	**Uralic**	Pagani *et al.* 2016
3	Khant1, Khant2, Khant3	**Khanty**	Russia	Siberia	>40	**Uralic**	Pagani *et al.* 2016
3	Evnk2, Evk14, Evk16	**Evenki**	Russia	Siberia	>40	**Altaic**	Pagani *et al.* 2016
3	Bur2, Bur6, Bur11	**Buryat**	Russia	Siberia	>40	**Altaic**	Pagani *et al.* 2016
3	YakS4, YakK1, YakK3	**Yakut**	Russia	Siberia	>40	**Altaic**	Pagani *et al.* 2016
3	EvenM1, EvenM2, EvenM3	**Even**	Russia	Siberia	>40	**Altaic**	Pagani *et al.* 2016

Note: the three Russian individuals come from three different subsets.

Supplementary Table S2. Ancient DNA samples used in this study.

Sample ID	Archaeological Culture	Date	Country	Region	Contributor
I1100	Anatolia_Neolithic	6500-6200 calBCE	Turkey	Barcın	Mathieson *et al.* 2015
I1102	Anatolia_Neolithic	6500-6200 calBCE	Turkey	Barcın	Mathieson *et al.* 2015
I1099	Anatolia_Neolithic	6500-6200 calBCE	Turkey	Barcın	Mathieson *et al.* 2015
I1103	Anatolia_Neolithic	6400-5600 calBCE	Turkey	Barcın	Mathieson *et al.* 2015
I1101	Anatolia_Neolithic	6400-5600 calBCE	Turkey	Barcın	Mathieson *et al.* 2015
I1097	Anatolia_Neolithic	6400-5600 calBCE	Turkey	Barcın	Mathieson *et al.* 2015
I0744	Anatolia_Neolithic	6400-5600 calBCE	Turkey	Barcın	Mathieson *et al.* 2015
I1579	Anatolia_Neolithic	6500-6200 calBCE	Turkey	Barcın	Mathieson *et al.* 2015
I1581	Anatolia_Neolithic	6500-6200 calBCE	Turkey	Barcın	Mathieson *et al.* 2015
I1096	Anatolia_Neolithic	6500-6200 calBCE	Turkey	Barcın	Mathieson *et al.* 2015
I1580	Anatolia_Neolithic	6500-6200 calBCE	Turkey	Barcın	Mathieson *et al.* 2015
I1098	Anatolia_Neolithic	6500-6200 calBCE	Turkey	Barcın	Mathieson *et al.* 2015
I1585	Anatolia_Neolithic	6500-6200 calBCE	Turkey	Barcın	Mathieson *et al.* 2015
I0708	Anatolia_Neolithic	6500-6200 calBCE	Turkey	Barcın	Mathieson *et al.* 2015
I0745	Anatolia_Neolithic	6500-6200 calBCE	Turkey	Barcın	Mathieson *et al.* 2015
I0746	Anatolia_Neolithic	6500-6200 calBCE	Turkey	Barcın	Mathieson *et al.* 2015
I1583	Anatolia_Neolithic	6500-6200 calBCE	Turkey	Barcın	Mathieson *et al.* 2015
I0707	Anatolia_Neolithic	6500-6200 calBCE	Turkey	Barcın	Mathieson *et al.* 2015
I0709	Anatolia_Neolithic	6500-6200 calBCE	Turkey	Barcın	Mathieson *et al.* 2015
I0725	Anatolia_Neolithic	6500-6200 calBCE	Turkey	Barcın	Mathieson *et al.* 2015
I0727	Anatolia_Neolithic	6500-6200 calBCE	Turkey	Barcın	Mathieson *et al.* 2015
I0724	Anatolia_Neolithic	6500-6200 calBCE	Turkey	Barcın	Mathieson *et al.* 2015
I0736	Anatolia_Neolithic	6500-6200 calBCE	Turkey	Barcın	Mathieson *et al.* 2015
I0723	Anatolia_Neolithic	6500-6200 calBCE	Turkey	Barcın	Mathieson *et al.* 2015

I0726	Anatolia_Neolithic	6500-6200 calBCE	Turkey	Barcın	Mathieson *et al*. 2015
I0231	Yamnaya	2910-2875 calBCE	Russia	Ekaterinovka, Southern Steppe, Samara	Haak *et al*. 2015
I0357	Yamnaya	3090-2910 calBCE	Russia	Lopatino I, Sok River, Samara	Haak *et al*. 2015
I0370	Yamnaya	3500-2700 calBCE	Russia	Ishkinovka I, Eastern Orenburg, Pre-Ural steppe, Samara	Haak *et al*. 2015
I0429	Yamnaya	3339-2917 calBCE	Russia	Lopatino I, Sok River, Samara	Haak *et al*. 2015
I0438	Yamnaya	3021-2635 calBCE	Russia	Luzkhi I, Samara River, Samara	Haak *et al*. 2015
I0439	Yamnaya	3305-2925 calBCE	Russia	Lopatino I, Sok River, Samara	Haak *et al*. 2015
I0441	Yamnaya	3010-2622 calBCE	Russia	Kurmanaevka III, Buzuluk, Samara	Haak *et al*. 2015
I0443	Yamnaya	3335-2912 calBCE	Russia	Grachevka II, Sok_River, Samara	Haak *et al*. 2015
I0444	Yamnaya	3335-2881 calBCE	Russia	Kutuluk I, Kutuluk River, Samara	Haak *et al*. 2015
RISE386	Sintashta	2298-2045 calBCE	Russia	Bulanovo	Allentoft *et al*. 2015
RISE391	Sintashta	2120-1887 calBCE	Kazakhstan	Tanabergen II	Allentoft *et al*. 2015
RISE392	Sintashta	2126-1896 calBCE	Russia	Stepnoe VII	Allentoft *et al*. 2015
RISE394	Sintashta	1949-1754 calBCE	Russia	Bulanovo	Allentoft *et al*. 2015
RISE395	Sintashta	1960-1756 calBCE	Russia	Bol'shekaraganskii	Allentoft *et al*. 2015

Supplementary Table S3. Human Origins data on present-day humans used in this study.

Sample ID	Population	Country	Region	Contributor
Nov_005	Nganasan	Russia	Central Asia Siberia	Lazaridis *et al*. 2014
ADR00514	Nganasan	Russia	Central Asia Siberia	Lazaridis *et al*. 2014
ADR00513	Nganasan	Russia	Central Asia Siberia	Lazaridis *et al*. 2014
ADR00509	Nganasan	Russia	Central Asia Siberia	Lazaridis *et al*. 2014
ADR00512	Nganasan	Russia	Central Asia Siberia	Lazaridis *et al*. 2014
ADR00504	Nganasan	Russia	Central Asia Siberia	Lazaridis *et al*. 2014
ADR00507	Nganasan	Russia	Central Asia Siberia	Lazaridis *et al*. 2014
ADR00511	Nganasan	Russia	Central Asia Siberia	Lazaridis *et al*. 2014
ADR00510	Nganasan	Russia	Central Asia Siberia	Lazaridis *et al*. 2014
ADR00508	Nganasan	Russia	Central Asia Siberia	Lazaridis *et al*. 2014
ADR00515	Nganasan	Russia	Central Asia Siberia	Lazaridis *et al*. 2014
HGDP00774	Han	China	East Asia	Patterson *et al*. 2012
HGDP00775	Han	China	East Asia	Patterson *et al*. 2012
HGDP00776	Han	China	East Asia	Patterson *et al*. 2012
HGDP00777	Han	China	East Asia	Patterson *et al*. 2012
HGDP00779	Han	China	East Asia	Patterson *et al*. 2012
HGDP00780	Han	China	East Asia	Patterson *et al*. 2012
HGDP00781	Han	China	East Asia	Patterson *et al*. 2012
HGDP00782	Han	China	East Asia	Patterson *et al*. 2012
HGDP00783	Han	China	East Asia	Patterson *et al*. 2012
HGDP00784	Han	China	East Asia	Patterson *et al*. 2012
HGDP00785	Han	China	East Asia	Patterson *et al*. 2012
HGDP00786	Han	China	East Asia	Patterson *et al*. 2012
HGDP00811	Han	China	East Asia	Patterson *et al*. 2012
HGDP00812	Han	China	East Asia	Patterson *et al*. 2012
HGDP00813	Han	China	East Asia	Patterson *et al*. 2012
HGDP00814	Han	China	East Asia	Patterson *et al*. 2012
HGDP00815	Han	China	East Asia	Patterson *et al*. 2012
HGDP00817	Han	China	East Asia	Patterson *et al*. 2012
HGDP00818	Han	China	East Asia	Patterson *et al*. 2012
HGDP00819	Han	China	East Asia	Patterson *et al*. 2012
HGDP00820	Han	China	East Asia	Patterson *et al*. 2012
HGDP00821	Han	China	East Asia	Patterson *et al*. 2012
HGDP00822	Han	China	East Asia	Patterson *et al*. 2012
HGDP00971	Han	China	East Asia	Patterson *et al*. 2012
HGDP00972	Han	China	East Asia	Patterson *et al*. 2012
HGDP00973	Han	China	East Asia	Patterson *et al*. 2012
HGDP00974	Han	China	East Asia	Patterson *et al*. 2012
HGDP00975	Han	China	East Asia	Patterson *et al*. 2012
HGDP00976	Han	China	East Asia	Patterson *et al*. 2012

HGDP00977	Han	China	East Asia	Patterson *et al*. 2012
HGDP01021	Han	China	East Asia	Patterson *et al*. 2012
HGDP01023	Han	China	East Asia	Patterson *et al*. 2012
HGDP01024	Han	China	East Asia	Patterson *et al*. 2012
HGDP00449	Mbuti	Congo	Africa	Patterson *et al*. 2012
HGDP00462	Mbuti	Congo	Africa	Patterson *et al*. 2012
HGDP00463	Mbuti	Congo	Africa	Patterson *et al*. 2012
HGDP00467	Mbuti	Congo	Africa	Patterson *et al*. 2012
HGDP00474	Mbuti	Congo	Africa	Patterson *et al*. 2012
HGDP00476	Mbuti	Congo	Africa	Patterson *et al*. 2012
HGDP00478	Mbuti	Congo	Africa	Patterson *et al*. 2012
HGDP00982	Mbuti	Congo	Africa	Patterson *et al*. 2012
HGDP00984	Mbuti	Congo	Africa	Patterson *et al*. 2012
HGDP01081	Mbuti	Congo	Africa	Patterson *et al*. 2012
HGDP00995	Karitiana	Brazil	America	Patterson *et al*. 2012
HGDP00999	Karitiana	Brazil	America	Patterson *et al*. 2012
HGDP01001	Karitiana	Brazil	America	Patterson *et al*. 2012
HGDP01003	Karitiana	Brazil	America	Patterson *et al*. 2012
HGDP01006	Karitiana	Brazil	America	Patterson *et al*. 2012
HGDP01010	Karitiana	Brazil	America	Patterson *et al*. 2012
HGDP01012	Karitiana	Brazil	America	Patterson *et al*. 2012
HGDP01013	Karitiana	Brazil	America	Patterson *et al*. 2012
HGDP01014	Karitiana	Brazil	America	Patterson *et al*. 2012
HGDP01015	Karitiana	Brazil	America	Patterson *et al*. 2012
HGDP01018	Karitiana	Brazil	America	Patterson *et al*. 2012
HGDP01019	Karitiana	Brazil	America	Patterson *et al*. 2012
Ul5	Ulchi	Russia	Central Asia Siberia	Rem Sukernik / Stanislav Dryomov
Ul31	Ulchi	Russia	Central Asia Siberia	Rem Sukernik / Stanislav Dryomov
Ul65	Ulchi	Russia	Central Asia Siberia	Rem Sukernik / Stanislav Dryomov
Ul6	Ulchi	Russia	Central Asia Siberia	Rem Sukernik / Stanislav Dryomov
Ul33	Ulchi	Russia	Central Asia Siberia	Rem Sukernik / Stanislav Dryomov
Ul71	Ulchi	Russia	Central Asia Siberia	Rem Sukernik / Stanislav Dryomov
Ul10	Ulchi	Russia	Central Asia Siberia	Rem Sukernik / Stanislav Dryomov
Ul43	Ulchi	Russia	Central Asia Siberia	Rem Sukernik / Stanislav Dryomov
Ul72	Ulchi	Russia	Central Asia Siberia	Rem Sukernik / Stanislav Dryomov
Ul44	Ulchi	Russia	Central Asia Siberia	Rem Sukernik / Stanislav Dryomov
Ul74	Ulchi	Russia	Central Asia Siberia	Rem Sukernik / Stanislav Dryomov
Ul19	Ulchi	Russia	Central Asia Siberia	Rem Sukernik / Stanislav Dryomov
Ul24	Ulchi	Russia	Central Asia Siberia	Rem Sukernik / Stanislav Dryomov
Ul59	Ulchi	Russia	Central Asia Siberia	Rem Sukernik / Stanislav Dryomov
Ul56	Ulchi	Russia	Central Asia Siberia	Rem Sukernik / Stanislav Dryomov
Ul55	Ulchi	Russia	Central Asia Siberia	Rem Sukernik / Stanislav Dryomov
Ul16	Ulchi	Russia	Central Asia Siberia	Rem Sukernik / Stanislav Dryomov

Ul69	Ulchi	Russia	Central Asia Siberia	Rem Sukernik / Stanislav Dryomov
Ul1	Ulchi	Russia	Central Asia Siberia	Rem Sukernik / Stanislav Dryomov
Ul36	Ulchi	Russia	Central Asia Siberia	Rem Sukernik / Stanislav Dryomov
Ul25	Ulchi	Russia	Central Asia Siberia	Rem Sukernik / Stanislav Dryomov
Ul52	Ulchi	Russia	Central Asia Siberia	Rem Sukernik / Stanislav Dryomov
Ul70	Ulchi	Russia	Central Asia Siberia	Rem Sukernik / Stanislav Dryomov
Ul51	Ulchi	Russia	Central Asia Siberia	Rem Sukernik / Stanislav Dryomov
Ul39	Ulchi	Russia	Central Asia Siberia	Rem Sukernik / Stanislav Dryomov
mixe0029	Mixe	Mexico	America	William Klitz / Cheryl Winkle
mixe0030	Mixe	Mexico	America	William Klitz / Cheryl Winkle
mixe0015	Mixe	Mexico	America	William Klitz / Cheryl Winkle
mixe0035	Mixe	Mexico	America	William Klitz / Cheryl Winkle
mixe0018	Mixe	Mexico	America	William Klitz / Cheryl Winkle
mixe0026	Mixe	Mexico	America	William Klitz / Cheryl Winkle
mixe0027	Mixe	Mexico	America	William Klitz / Cheryl Winkle
mixe0028	Mixe	Mexico	America	William Klitz / Cheryl Winkle
mixe0007	Mixe	Mexico	America	William Klitz / Cheryl Winkle
mixe0009	Mixe	Mexico	America	William Klitz / Cheryl Winkle

Supplementary Table S4. Statistics of the *qpAdm* models.

Test	Outgroup set	Nganasan	Yamnaya	Anatolia	chi-square
Khanty	Han, Mbuti, Karitiana, Ulchi and Mixe	0.521	0.479	0	10.056
Maris	Han, Mbuti, Karitiana, Ulchi and Mixe	0.281	0.465	0.254	10.493
Udmurts	Han, Mbuti, Karitiana, Ulchi and Mixe	0.261	0.611	0.128	9.032
Iranians	Han, Mbuti, Karitiana, Ulchi and Mixe	0.016	0.141	0.843	7.053
Finns	Han, Mbuti, Karitiana, Ulchi and Mixe	0.101	0.589	0.31	6.623
Estonians	Han, Mbuti, Karitiana, Ulchi and Mixe	0.04	0.568	0.391	8.095
Hungarians	Han, Mbuti, Karitiana, Ulchi and Mixe	0.032	0.412	0.556	8.398
Russian North	Han, Mbuti, Karitiana, Ulchi and Mixe	0.144	0.571	0.284	2.255
Russian	Han, Mbuti, Karitiana, Ulchi and Mixe	0.042	0.517	0.441	5.389
Russian West	Han, Mbuti, Karitiana, Ulchi and Mixe	0.035	0.526	0.440	0.819
Croats	Han, Mbuti, Karitiana, Ulchi and Mixe	0.042	0.303	0.655	4.373
Germans	Han, Mbuti, Karitiana, Ulchi and Mixe	0.027	0.373	0.6	5.999
Poles	Han, Mbuti, Karitiana, Ulchi and Mixe	0.023	0.561	0.416	2.634

© 2020 by the authors. Licensee MDPI, Basel, Switzerland. This article is an open access article distributed under the terms and conditions of the Creative Commons Attribution (CC BY) license (http://creativecommons.org/licenses/by/4.0/).
